# Supplementary material for: Discovery and pharmacophoric characterization of chemokine network inhibitors using phage-display, saturation mutagenesis and computational modelling
Source: Nat Commun. 2023 Sep 16;14:5763. doi: 10.1038/s41467-023-41488-z (PMC10505172; doi:10.1038/s41467-023-41488-z)
Supplement: Supplementary file 4 — Source Data [file 41467_2023_41488_MOESM4_ESM.zip › SourceData/scripts/xngs_20230809084627.tar!/xngs/analysis/SV_EVAA7_tree.pdf]

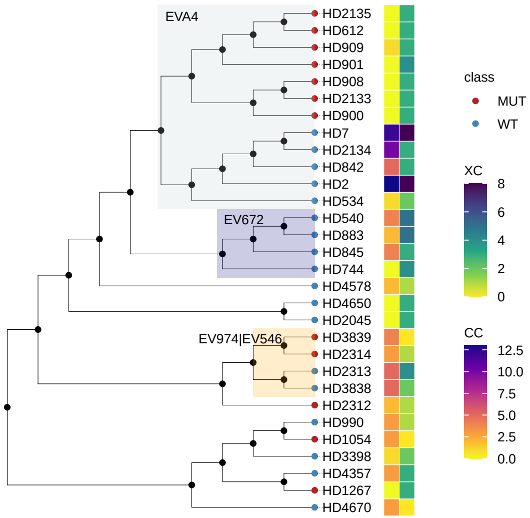
EVA4
EV672
EV974|EV546
HD2135
HD612
HD909
HD901
HD908
HD2133
HD900
HD7
HD2134
HD842
HD2
HD534
HD540
HD883
HD845
HD744
HD4578
HD4650
HD2045
HD3839
HD2314
HD2313
HD3838
HD2312
HD990
HD1054
HD3398
HD4357
HD1267
HD4670
class
MUT
WT
XC
8
6
4
2
0
CC
12.5
10.0
7.5
5.0
2.5
0.0
